# P-831. Association Between Adherence of Antibiotic Practice with a Bacterial/Viral Test Result and Outcomes in US ED/UC

**DOI:** 10.1093/ofid/ofaf695.1039

**Published:** 2026-01-11

**Authors:** Lior Kellerman, Tanya Gottlieb, Boris Lebedenko, Roy Navon, Brian DuChateau, Gabriel A Bien-Willner

**Affiliations:** MeMed, Haifa, Israel, Haifa, Hefa, Israel; MeMed, Tirat Carmel, HaZafon, Israel; MeMed Diagnostic, Tirat Carmel, Hefa, Israel; MeMed Diagnostics, Tirat Carmel, HaZafon, Israel; MeMed Diagnostic, Tirat Carmel, Hefa, Israel; bwgprecision medicine, Conroe, Texas

## Abstract

**Background:**

Difficulty in determining the etiology of infections in the emergency department (ED) and urgent care (UC) settings often leads to suboptimal outcomes.

MeMed BV® (MMBV) is an FDA-cleared host-protein test that differentiates bacterial and viral infections, with sensitivity and specificity >90% and negative predictive value >98%.

This post hoc analysis evaluated whether alignment between MMBV and antibiotic prescribing improved outcomes. The hypothesis was that viral MMBV cases untreated with antibiotics have similar outcomes to those treated, while untreated bacterial cases have worse outcomes. We also estimated potential impact on costs.

**Figure:**
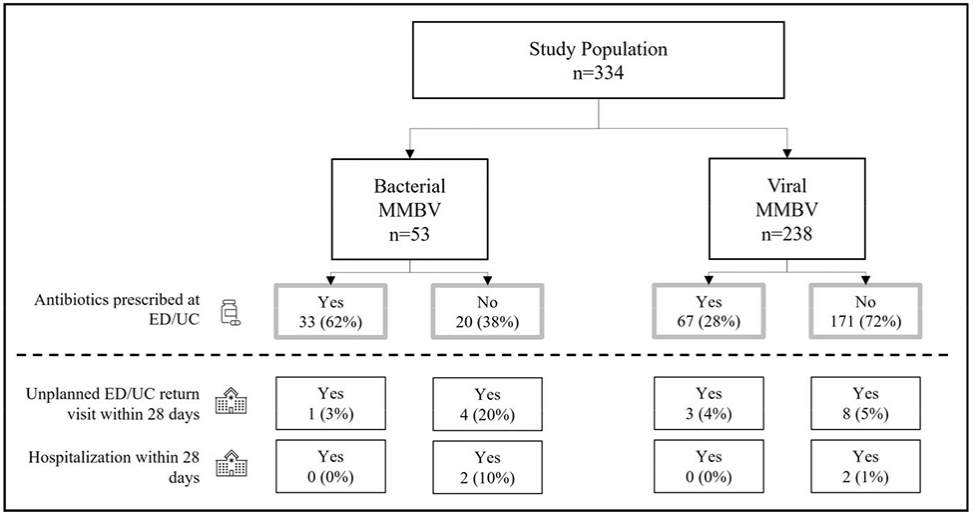
Alignment between antibiotic prescription and MeMed BV (MMBV) results and outcomes

This project has been supported in part with federal funds from the Department of Health and Human Services; Administration for Strategic Preparedness and Response; Biomedical Advanced Research and Development Authority (BARDA), under contract number 75A50123C00041.

**Methods:**

US adults (≥18) from 11 sites, with suspected acute infections from two prospective studies (NCT04690569, NCT05762302) were included. Only those discharged with complete follow-up were analyzed.

MMBV scores range 0-100: < 35 indicate a viral (or non-bacterial) infection, 35–65 is equivocal, and >65 indicates a bacterial infection (or co-infection). Based on MMBV’s validated accuracy, antibiotics were considered unwarranted for MMBV < 35 and warranted for MMBV >65.

Outcomes included unplanned ED/UC return visits and hospitalization in 28 days. Cost analysis used 2024 AHRQ Healthcare Cost & Utilization Project data.

**Results:**

The analysis included 334 adults; 60% female, median age 36 years (IQR: 27-49), 48% ED enrollment.

Patients with MMBV < 35 (n=238) not prescribed antibiotics showed no significant difference in ED/UC return visits (5% vs. 4%; p=0.947) or hospitalization (1% vs. 0%; p=0.375) as compared to patients prescribed antibiotics.

Patients with MMBV >65 (n=53) not prescribed antibiotics had significantly more ED/UC return visits (20% vs. 3%; p=0.042) and increased hospitalization (10% vs. 0%; p=0.067) as compared to patients prescribed antibiotics.

In the MMBV-aligned group (n=204), there were 9 return visits, 2 hospitalizations, and 4 hospital days, costing $16,611 ($81/patient). In the non-aligned group (n=87), there were 7 return visits, 2 hospitalizations, and 31 hospital days, costing $73,327 ($843/patient), providing $761/patient savings with MMBV alignment.

**Conclusion:**

Alignment between antibiotic prescribing and MMBV results is associated with better patient outcomes and lower hospital costs.

**Disclosures:**

Lior Kellerman, M.D, MeMed Diagnostic: Employee Tanya Gottlieb, PhD, MeMed Diagnostic: Employee Boris Lebedenko, MSc, MeMed Diagnostic: Employee Roy Navon, MSc, MeMed Diagnostic: Employee Brian DuChateau, PhD, MeMed Diagnostic: Employee Gabriel A. Bien-Willner, MD, PhD, FCAP, MeMed Diagnostic: Advisor/Consultant

